# Reduction in skeletal muscle fibrosis of spontaneously hypertensive rats after laceration by microRNA targeting angiotensin II receptor

**DOI:** 10.1371/journal.pone.0186719

**Published:** 2017-10-23

**Authors:** Roberta Sessa Stilhano, Vivian Yochiko Samoto, Leonardo Martins Silva, Gustavo José Pereira, Adolfo Garcia Erustes, Soraya Soubhi Smaili, Sang Won Han

**Affiliations:** 1 Department of Biophysics, Escola Paulista de Medicina, Universidade Federal de Sao Paulo (UNIFESP), Sao Paulo, Brazil; 2 Department of Pharmacology, Escola Paulista de Medicina, Universidade Federal de Sao Paulo (UNIFESP), Sao Paulo, Brazil; Universidade Federal do Rio de Janeiro, BRAZIL

## Abstract

Regeneration of injured skeletal muscles is affected by fibrosis, which can be improved by the administration of angiotensin II (AngII) receptor (ATR) blockers in normotensive animals. However, the role of ATR in skeletal muscle fibrosis in hypertensive organisms has not been investigated yet. The *tibialis anterior* (TA) muscle of spontaneously hypertensive (SHR) and Wistar rats (WR) were lacerated and a lentivector encoding a microRNA targeting AngII receptor type 1 (*At1*) (Lv-mirAT1a) or control (Lv-mirCTL) was injected. The TA muscles were collected after 30 days to evaluate fibrosis by histology and gene expression by real-time quantitative PCR (RT-qPCR) and Western blot. SHR’s myoblasts were analyzed by RT-qPCR, 48 h after transduction. In the SHR’s TA, AT1 protein expression was 23.5-fold higher than in WR without injury, but no difference was observed in the angiotensin II receptor type 2 (AT2) protein expression. TA laceration followed by suture (LS) produced fibrosis in the SHR (23.3±8.5%) and WR (7.9±1.5%). Lv-mirAT1 treatment decreased *At1* gene expression in 50% and reduced fibrosis to 7% 30 days after. RT-qPCR showed that reduction in *At1* expression is due to downregulation of the *At1a* but not of the *At1b*. RT-qPCR of myoblasts from SHR transduced with Lv-mirAT1a showed downregulation of the *Tgf-b1*, *Tgf-b2*, *Smad3*, *Col1a1*, *and Col3a1* genes by mirAT1a. In vivo and in vitro studies indicate that hypertension overproduces skeletal muscle fibrosis, and AngII-AT1a signaling is the main pathway of fibrosis in SHR. Moreover, muscle fibrosis can be treated specifically by *in loco* injection of Lv-mirAT1a without affecting other organs.

## Introduction

Skeletal muscles weight about 33–40% of total body weight of non-obese men and women, being responsible for posture and voluntary movements [1]. Muscle injury can cause muscle contracture, atrophy, and fibrotic scar tissue, which can affect physical movements [2,3]. Complete muscle recovery after a serious injury is usually affected by the establishment of a fibrotic scar tissue at the site of injury. Sportsmen who demand highly efficient muscle functioning can suffer with even small fibrosis, and recurrent injuries can occur at the same site during physical exercises [4,5].

Fibrosis is part of the final step of muscle recovery and is characterized by intense synthesis of extracellular matrix (ECM) proteins by myofibroblasts, which results in the formation of connective tissue scar [6]. In cases of severe muscle injury, fibrogenesis is exacerbated and results in the formation of a dense scar, besides irregular and malfunctioning tissue [3,7]. Therapy based on anti-inflammatory drugs, ice, and rest have limited efficacy in preventing or treating muscle fibrosis after injury [8,9].

Several studies have shown that hypertension promotes fibrosis, and angiotensin II (AngII) receptor type 1 (AT1) is the key factor in hypertension and fibrosis [10,11]. Reduction of fibrosis by AT1 blockers is an important evidence of the fibrotic activity of AngII-AT1[12–14]. Even in the chronic muscular genetic diseases, such as Marfan syndrome and Duchenne muscular dystrophy, the AT1 antagonist losartan was able to reduce fibrosis, improving skeletal muscle regeneration [13].

TGF-β1 is a pleiotropic factor that orchestrate various cell activities. It promotes the negative proliferation of myeloid, epithelial, and lymphoid tumor cells [15,16], but it also stimulates fibroblast proliferation [17] and ECM synthesis in myofibroblasts [18]. Due to these last activities, TGF-β1 is considered the most important fibrotic factor [19,20]. It is known that AngII-AT1 and TGF-β1 signaling cross-talk activates Smad2/3 and upregulates the fibrogenic factor connective tissue growth factor (CTGF) and collagen I expression [21]. However, the pro-fibrotic AngII-AT1 activity can also occur via TGF-β1 in an independent manner [22,23].

According to the World Health Organization (http://www.who.int), there are about 1 billion hypertensive people in the world. In addition, hypertension is one of the most important risk factors for cardiovascular diseases, which affect heart, brain, peripheral limbs, and other organs [24,25]. Although there are several significant numbers of studies showing the effect of AngII-AT1 signaling in skeletal muscle fibrosis, the knowledge about this signaling in skeletal muscles of hypertensive organisms is not clear [14,26–28].

The spontaneously hypertensive rat (SHR) has been largely used to study physiology and pathophysiology related to human hypertension (over 13,000 Medline references were found using both “SHR” and “hypertension” words) due to the genetic predisposition to hypertension and age dependence to manifest hypertension, as occurs in humans [29]. The spontaneous cardiac fibrosis is a major complication of hypertension in SHR, and the development of fibrous tissue is related to overexpression of TGF-β1, which is stimulated by AngII [30–32]. The AT1 blockers or angiotensin converting enzyme (ACE) inhibitors have been successfully used to reduce hypertrophy and cardiac fibrosis in this model [33–35]. Although the AT1 blocker losartan showed significant benefits in the treatment of muscle fibrosis, this medication has a systemic action and can cause several adverse effects [36–38]. Therefore, the search for a localized therapy for patients with muscle injury and fibrosis is necessary.

Here, we report a study on the relationship between fibrogenesis and AT1 in the skeletal muscle of SHR after a severe injury, using the normotensive Wistar rat (WR) as a control. To establish a deep injury in the skeletal muscle, we chose laceration followed by suture because it is a common practice among orthopedists to treat deep muscle injury, which can occur among sportsmen and patients who suffered of vehicle accidents. In addition, as it was commented by Bedair et al [14], laceration is a highly reproducible muscle injury model, its histopathology is very similar to that observed in strain injury patterns, and any treatment benefits seen in this model would likely bring equal or greater benefit in less severe strain injuries. We hypothesized that the skeletal muscle of SHR should express more ATR and, should thus produce more fibrotic tissue after injury. Down-regulation of the AT1 receptor in the SHR muscle by miRNA should reduce fibrosis and promote better muscle regeneration. This strategy may provide a useful *in loco* treatment of patients with deep muscle injuries.

## Materials and methods

### Animals and muscle laceration

The SHR were obtained from the animal facility of the Institute of Biomedical Sciences, University of São Paulo (USP), and WR were obtained from the National Institute of Pharmacology, Federal University of São Paulo (UNIFESP). All rats (35 SHR and 15 WR) were 16-week-old males (weight: 300–350 g). All procedures involving animals were performed only after the project protocol received approval by the Research Ethics Committee of UNIFESP (Approval number: 0347/12). The completed ARRIVE (Animal Research: Reporting In Vivo Experiments) Checklist is in S1 File.

**Fig 1:**
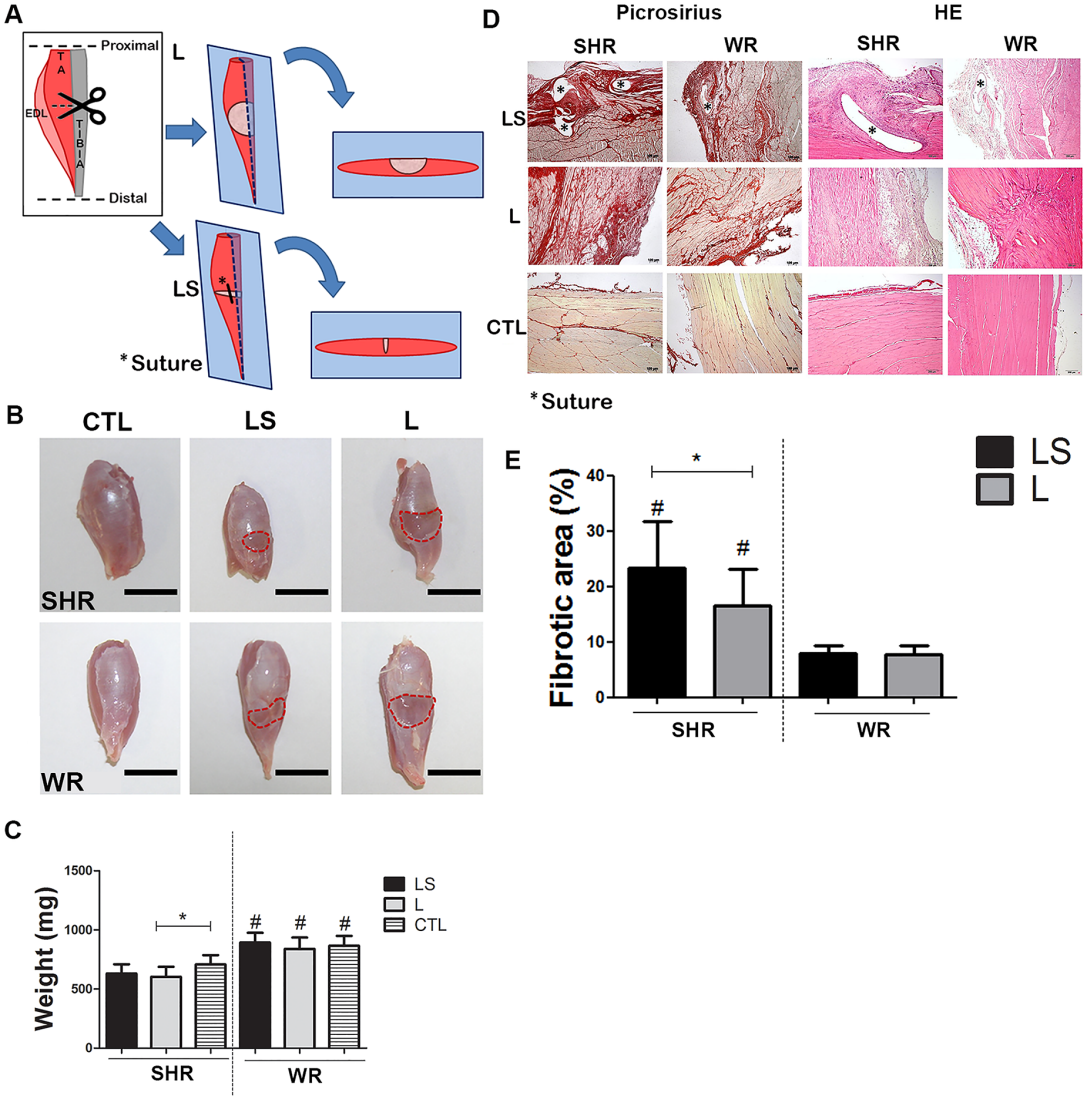
Rat muscle injury model. (A) Schematic design of the rat muscle injury model. The *tibialis anterior* (TA) muscle was cut in the middle (laceration group: L) and sutured (laceration-suture group: LS) or not. Four weeks after this procedure, the muscle was excised and cut longitudinally to histology. (B) Macroscopic images of the TA muscle from SHR and WR (scale bar = 1 cm). (C) TA muscle weight comparison among groups Representative micrographs of sections stained with PR or HE (scale bar = 50 μm). (D) Fibrotic areas quantified using images from Fig 1D (E). * indicates statistically significant differences (p<0.05) when animals of the same species of rats were compared with subjected to different laceration processes. ^#^ indicates statistically significant differences (p<0.05) when animals of different species were compared for subjected to the same laceration process. Values represent mean ± standard deviation. n = 5 rats per group. TA: *tibialis anterior*; EDL: *extensor digitorum longus*; SHR: spontaneously hypertensive rat; WR: Wistar rat; CTL: control group (without injury); PR: Picrosirius Red; HE: hematoxylin-eosin.

For muscle laceration, SHR and WR were initially anesthetized with intraperitoneal (ip) injection of ketamine (40 mg/kg) and xylazine (20 mg/kg). Muscles were surgically lacerated as previously described [39], with some modifications. Briefly, the tibialis anterior (TA) muscle from both legs were exposed and transversely cut with a scissor in the middle of the muscle (Fig 1A). The muscles were sutured (laceration-suture group, LS) or not (laceration group, L) after the injury. In the LS group, a PDSII 5–0 suture (Ethicon; NJ, USA) was placed at the medial edge of the lacerated site. After laceration, the skin was closed with black silk 4–0 suture (Ethicon). Four weeks after laceration, all animals were euthanized to evaluate muscle healing and fibrosis. Five muscles from each group were evaluated by histology and other five muscles were evaluated by real-time quantitative PCR (RT-qPCR) and Western blot.

### Primary muscle cell culture from SHR and WR

Primary muscle cells were obtained by enzymatic disaggregation of leg muscle from 1-day-old SHR (CEDEME animal facility, UNIFESP) or WR (INFAR animal facility, UNIFESP) and cultured as previously described previously [40–42]. Eight rats were used in this study. Isolated cells were then suspended in Dulbecco’s modified Eagle’s medium (DMEM; Life Technologies; NY, USA) with 10% fetal bovine serum (FBS; Life Technologies), 10% horse serum (Sigma Chemical Co.; MO, USA), 0.5% chick embryo extract (United States Biological; MA, USA), penicillin/streptomycin (100 U/mL; Life technologies) and 2 mM L-glutamine (Life technologies), and maintained at 37°C with 5% CO_2_. Cells were then plated in collagen-coated flasks. Two hours after plating, the supernatant was removed from the flask and replated in a fresh collagen-coated flask. Within 2-h incubation, mostly fibroblasts adhered to the collagen coat. The serial replating of non-adherent cells was repeated daily for 4 days to establish a primary myoblast cell culture [40,41]. The rat skeletal muscle cell line L6 (ATCC; VA, USA) was used as a control.

### AngII effect in primary muscle cells

SHR and WR primary muscle cells were seeded on a 24-well plate (3x10^4^ cells/well) and 24 hours later AngII (kindly donated by Dr. Julie Bossuyt, University of California, Davis) was added to a final concentration of 100 nM/well [43,44]. After 24 hours, the total RNA was extracted and genes were quantified following methods described in the Gene expression analysis by Real-time quantitative PCR (RT-qPCR) section.

### Lentivector construction, production, and transduction

The mirAT1a (micro RNA to AT1a receptor) and mirCTL (micro RNA control) sequences are the same as those from Fan et al [45]. They were obtained from GenScript (NJ, USA) and were cloned into the pUC57 vector. The mirAT1a and mirCTL sequences were removed from the pUC57 vector using MluI/ClaI and cloned into the pLVTHM vector (Addgene; MA, USA), which was previously treated with the same enzymes. These vectors were named as pLv-mirAT1a and pLv-mirCTL. All plasmids were amplified using the Plasmid Midi Kit^®^ (Qiagen; Hilden, Germany) and sequenced in the 3100 Avant Genetic Analyzer (Life Technologies).

The lentivectors were produced using the plasmids psPAX2 (Addgene), M5 (Express VSV-G; this vector was kindly provided by Prof. D.von Laer; Chemotherapeutisches Forschungsinstitut, Georg-Speyer-Haus). Lentivector production, concentration, and titration were performed following the protocol established by Naldini et al [46]. In brief, lentivectors were produced by transfection of human embryonic kidney cells (HEK-293T), which were cultured (37°C; 5% CO_2_) in DMEM (Life Technologies) with 10% FBS (Life Technologies), penicillin/streptomycin (100 U/mL; Life technologies), and 2 mM L-glutamine (Life technologies). The culture supernatant was collected after 2 and 3 days, and filtered through a 0.45-μm pore diameter (Millipore; NY, USA) syringe filter, and concentrated (37,000 xg; 4°C; 2 h) in a Sorvall (5C plus model; Thermo Fisher Scientific; MA, USA) centrifuge. Lentivectors produced with pLv-mirAT1a and pLv-mirCTL were named as Lv-mirAT1a and Lv-mirCTL, respectively.

To determine the viral titer, which was expressed as transducing units (TU) per mL, HEK-293T cells were transduced with different concentrations of lentiviral vectors in the presence of Polybrene (8 μg/mL; Sigma). After 3 days, the HEK-293T cells expressing GFP were counted (>10,000 events analyzed) using a FACScan (Becton Dickinson, BD; CA, USA) cytometer and the data was analyzed using the FlowJo (TreeStar Inc.; OR, USA) software. The titer is given by GFP positive cells (%) X total number of cells on the day of transduction / volume of virus.

For transduction of L6 and rat primary muscle cells from SHR (passage 4), these cells were seeded on a 6-well plate at 70% confluence, with DMEM supplemented with 10% FBS. After 24 h, the medium was replaced with a fresh one containing Polybrene (8 μg/mL; Sigma), and Lv-mirAT1a or Lv-mirCTL vectors were added to the media with multiplicity of infection (MOI) to transduce L6 (MOI = 10) or rat primary muscle cells (MOI = 50). After 24 h, all media were replaced with fresh ones.

### Gene therapy

The SHR and WR were anesthetized with ip injection of ketamine (40 mg/kg) and xylazine (20 mg/kg), and muscles were lacerated as described above. Soon after laceration, 1.2 x 10^7^ TU of Lv-mirAT1a or Lv-mirCTL in DMEM (100 μL) were injected in each TA muscle. Four weeks after the treatment, the muscles were removed for histology, gene, and protein expression analysis.

### Histological analysis

After the TA muscles were excised, they were weighted and fixed in formaldehyde (10%; 24 h). Next, the muscles were cut (Fig 1A), dehydrated, and embedded in paraffin. Four-micrometer sections were obtained and stained with hematoxylin-eosin (HE) or Picrosirius Red (PR).

Digital images were acquired using a microscope, and 20 fields per slide were analyzed using the Image Pro Plus (Media Cybernetics, Inc.; MD, USA) software. Fibrotic areas were expressed in percentage of PR stained area per total injured area, constituted by the middle portion of TA where the injury was established.

### Gene expression analysis by real-time quantitative PCR (RT-qPCR)

Total RNA from TA muscles, heart, primary culture of muscle cells, or L6 cells were extracted with the Trizol reagent (Life Technologies) and treated with DNAse I (Invitrogen). Complementary DNA (cDNA) was synthetized using RNA (1μg) with the High-Capacity cDNA Reverse Transcription (Life Technologies) kit. The real-time quantitative PCR RT-qPCR was performed using the QuantiFast Probe Assay (Qiagen) kit or SYBR green kit (Qiagen) in the Rotor Gene-Q (Qiagen). The QuantiFast Probe Assay kit was used to quantify rat Angiotensin II receptor, type 1a (*Agtr1a*; Qiagen, Catalog number (Cat.N°): QF00333130), Angiotensin II receptor, type 1b (*Agtr1b*; Qiagen, Cat.N°: QF00146503), Angiotensin II receptor, type 2 (*Agtr2*; Qiagen, Cat.N°: QF00267043) and the control gene peptidylprolyl isomerase A (*Ppia*; Qiagen, Cat.N°: QF00531300). The SYBR green kit was used to quantify the expression of the following genes: Transforming growth factor, beta 1 (*Tgfb1*); collagen, type I, alpha 1 (*Col1a1*); collagen, type III, alpha 1 (*Col3a1*); connective tissue growth factor (*Ctgf*); SMAD family member 2 (*Smad2*); SMAD family member 3 (*Smad3*); transforming growth factor, beta receptor 1 (*Tgfbr1*); transforming growth factor, beta receptor 2 (*Tgfbr2*); and 18S ribosomal RNA (*Rn18S*). The primer sequences used in the real-time RT-qPCR were described in the S1 Table. The relative gene expression was calculated by the 2^-ΔΔCT^ method. Changes in mRNA expression were expressed as fold-changes relative to a control, which was specified in the figure legends.

### Gene expression analysis by Reverse Transcription-PCR (RT-PCR)

The RT-PCR was performed using the Platinum TAQ DNA polymerase (Life Technologies) kit according to the manufacturer’s instructions. The genes evaluated by the RT-PCR were as follows: myogenic factor 5 (*Myf5*), myogenic regulatory factor (*Myod*), and desmin (*Des*). The primer sequences were obtained using the Primer 3 (University of Massachusetts, USA) software, and the sequences are listed in the S1 Table.

### Protein analysis

Samples from the TA muscle (SHR: 600–700 mg; WR: 800–900 mg) or heart (1.0–1.2 mg) were excised and crushed in the Ultra-Turrax (IKA; Campinas, Brazil) tissue triturator with extraction buffer (50 mM Tris, 150 mM NaCl, 0.05% deoxycholate, 1% NP-40, 1.0 mM EDTA, 0.1% SDS; pH 8.0; 1.0 mL) containing phenylmethylsulfonyl fluoride (PMSF; 1/100, v/v). The samples were centrifuged (8000 xg; 4°C; 5 min) after the supernatant was collected and stored (-80°C) until analysis. Total protein content was quantified using the Bio-Rad DC protein assay (Bio-Rad; CA, USA) kit. The samples were incubated (95°C; 5 min) with sample buffer (375 mM Tris/HCl, 120 mM EDTA, 12% SDS, 1.0 mg/mL bromophenol blue, 40% glycerol; pH 6.8).

For the western blot analysis, protein aliquots (50 μg) from each sample were mixed with a loading buffer (375 mM Tris-HCl, 120 mM EDTA, 12% SDS, 1.0 mg/mL bromophenol blue, 40% glycerol; pH 6.8) and loaded on 12% SDS-PAGE gel for electrophoresis (100 V; 2 h). After this run was completed, the gel contents were electrotransferred (100 V; 1 h; RT) onto a nitrocellulose membrane (0.45-μm pore size; Hybond ECL; Amersham Biosciences; Freiburg, Germany). The membrane was blocked (2 h; RT) in Tris-buffered saline (TBS; 10.0 mM Tris, 150 mM NaCl; pH 7.5) containing Tween 20 (TBS-T; 0.2%) and nonfat dry milk (5%). Next, the membranes were incubated (4°C; overnight) with primary antibodies in nonfat dry milk (2%). The antibodies were as follows: anti-AT1 (Santa Cruz Biotechnology; TX, USA; mouse monoclonal antibody; Cat. No. sc-81671; 1:200 dilution), anti-AT2 (Santa Cruz Biotechnology; rabbit polyclonal antibody; Cat. No. sc-9040; 1:200 dilution), or anti-ß actin (Fermentas; rabbit polyclonal antibody; Cat. No. RB-9421; 1:1000 dilution). The membranes were washed with TBS-T and incubated (2 h; RT) with peroxide-conjugated secondary antibody (Pierce Biotechnology; IL, USA) containing nonfat dry milk (2%), washed with TBS-T, and incubated (5 min) with Western Lightning^®^ Plus-ECL (PerkinElmer; MA, USA). The labeled bands were captured in the Alliance 4.7, (Uvitec; Cambridge, UK) scan. Membranes were stripped and relabeled with anti-ß-actin antibody to capture the bands again. The pixels of each band were measured using the Image J (v. 1.47; NIH; MD, USA) software.

### Statistical analysis

All statistical analyses were performed using Student t-tests (two-tail comparisons) or one-way analysis of variance (ANOVA) with post hoc Bonferroni’s test using Prism 6 software (Graphpad; CA, USA). Differences between conditions were considered significant if P < 0.05.

## Results

### Quantitative histological analysis of muscle fibrosis after laceration

To study the relationship between hypertension and fibrosis after muscle injury, two muscle injury methods were first compared: Laceration (L) and Laceration followed by Suture (LS) (Fig 1A). Four weeks after the L and LS procedures were performed in SHR and WR, the muscles were isolated for analyses. Macroscopic analysis showed that animals in the L group exhibited a bigger injured area as compared to those in the LS group (Fig 1B). The TA muscles from WR were 18.2% heavier than those from SHR (Fig 1C). Laceration caused a significant (15%) decrease in the TA muscle weight in the SHR/L group as compared to that in the SHR/CTL group, however, in the WR/L group the TA muscle weight was not significantly different in comparison to the non-lacerated group WR/CTL (Fig 1C). Tissue fibrosis, as assessed by PR staining, showed that fibrosis was clearly established after four weeks in both injury models. The laceration area was characterized by an intense reddish staining (Fig 1D). The cells in the scar were more disorganized in animals of the L group as compared to those in animals of the LS group. The animals in the SHR/LS group showed a significant increase in the fibrotic area as compared to those in the WR/LS group (Fig 1E). However, no difference was observed in the fibrotic area between the LS and L groups, within the WR group.

### Expression of AngII receptors in the skeletal and cardiac muscles of hypertensive and normotensive rats

**Fig 2:**
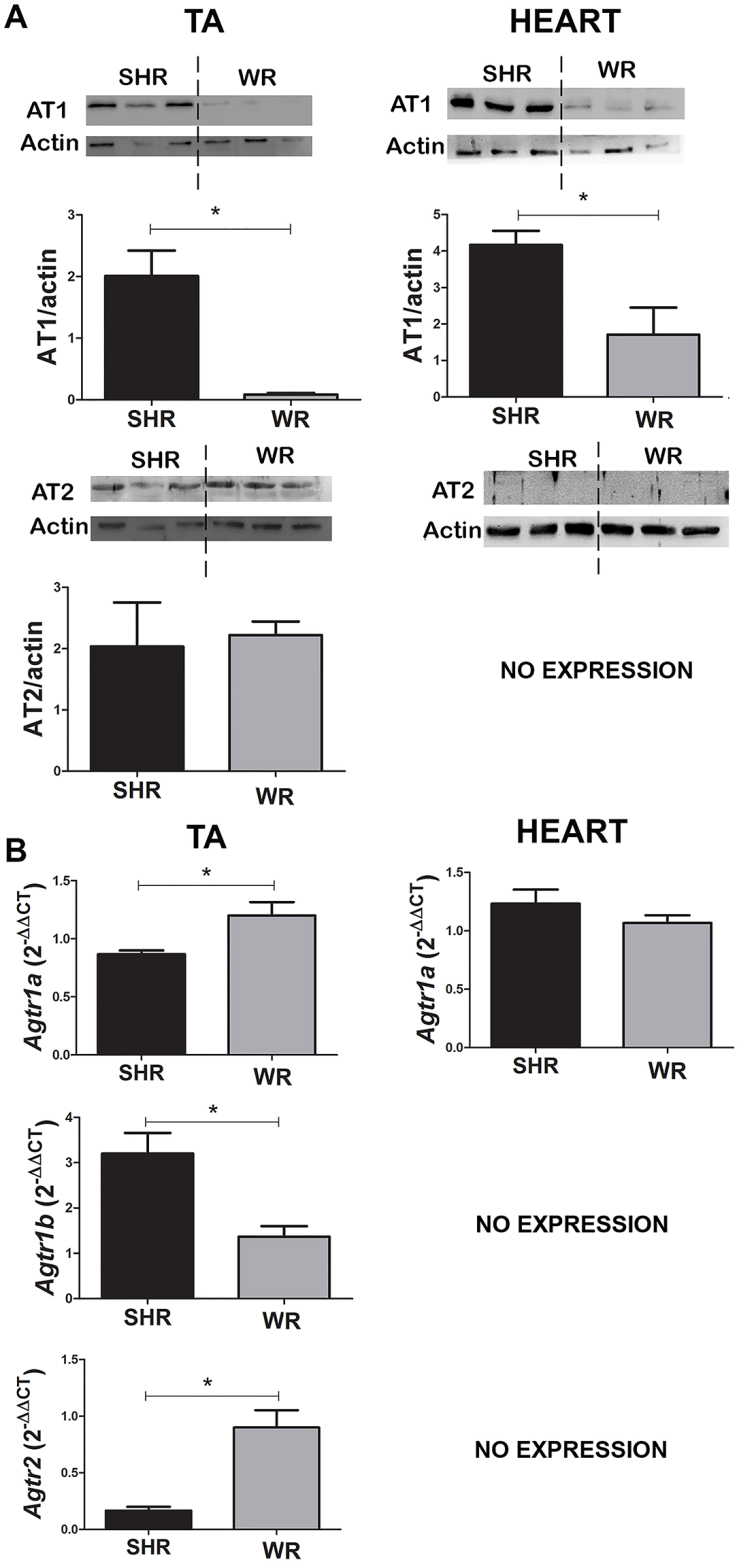
Expression of AngII receptors in the skeletal and cardiac muscles. (A) The AT1 and AT2 receptors in the TA and heart muscles from animals of the SHR and WR groups were quantified by Western Blot analysis. Band intensities were quantified by the Image J program and the ATR/β-actin ratios were plotted (n = 3 rats per group). In the heart, AT2 receptor expression was not detected in both rat strains. (B) Expression of the *Agtr1a*, *Agtr1b*, and *Agtr2* genes in the TA and heart muscles from animals of the SHR and WR groups was quantified by RT-qPCR (n = 5 rats per group). Expression of the *Agtr1b* and *Agtr2* genes was not detected in the heart from both rat strains. * p<0.05. TA: *tibialis anterior*. SHR: spontaneously hypertensive rat. WR: Wistar rat.

The expression of AT1 and AT2 receptor proteins in the TA and heart muscles from animals of the of the SHR and WR groups without injury was quantified by Western blot analysis (Fig 2A) to correlate increase in fibrosis in animals of the SHR group with expression of AT1 receptors. As compared to animals of the WR group, those in the SHR group showed a much higher AT1 expression in the TA muscle (25-fold) and heart (2.4-fold). Regarding the AT2 receptor, no gene or protein expression was detected in the SHR and WR hearts. In the TA muscle, the AT2 receptor gene expression in the WR group was about 2-fold higher than SHR, however no difference was observed between SHR and WR groups in terms of protein.

We also compared gene expression of AngII receptors in the TA muscle and heart of animals of the SHR and WR groups by RT-qPCR (Fig 2B). In the TA muscle, animals of the WR group showed a significantly higher expression of *Agtr1a* (1.4-fold) in comparison to those of the SHR group. However, expression of *Agtr1b* in animals of the SHR group expressed 2.4-fold higher than in those of the WR group. Therefore, the higher expression of AT1 receptor protein by animals of the SHR group should be of the *Agtr1b* gene expression. On the other side, *Agtr2* gene expression in animals of the WR group was higher (5.6-fold).

In the heart, no difference was observed in the *Agtr1a* gene expression between animals of the SHR and WR groups (Fig 2B). The *Agtr1b* gene was expressed only in animals of the WR group, and the *Agtr2* gene was not expressed in any of the rats.

### Fibrogenic gene expression profile in SHR and WR myoblasts after treatment with AngII

**Fig 3:**
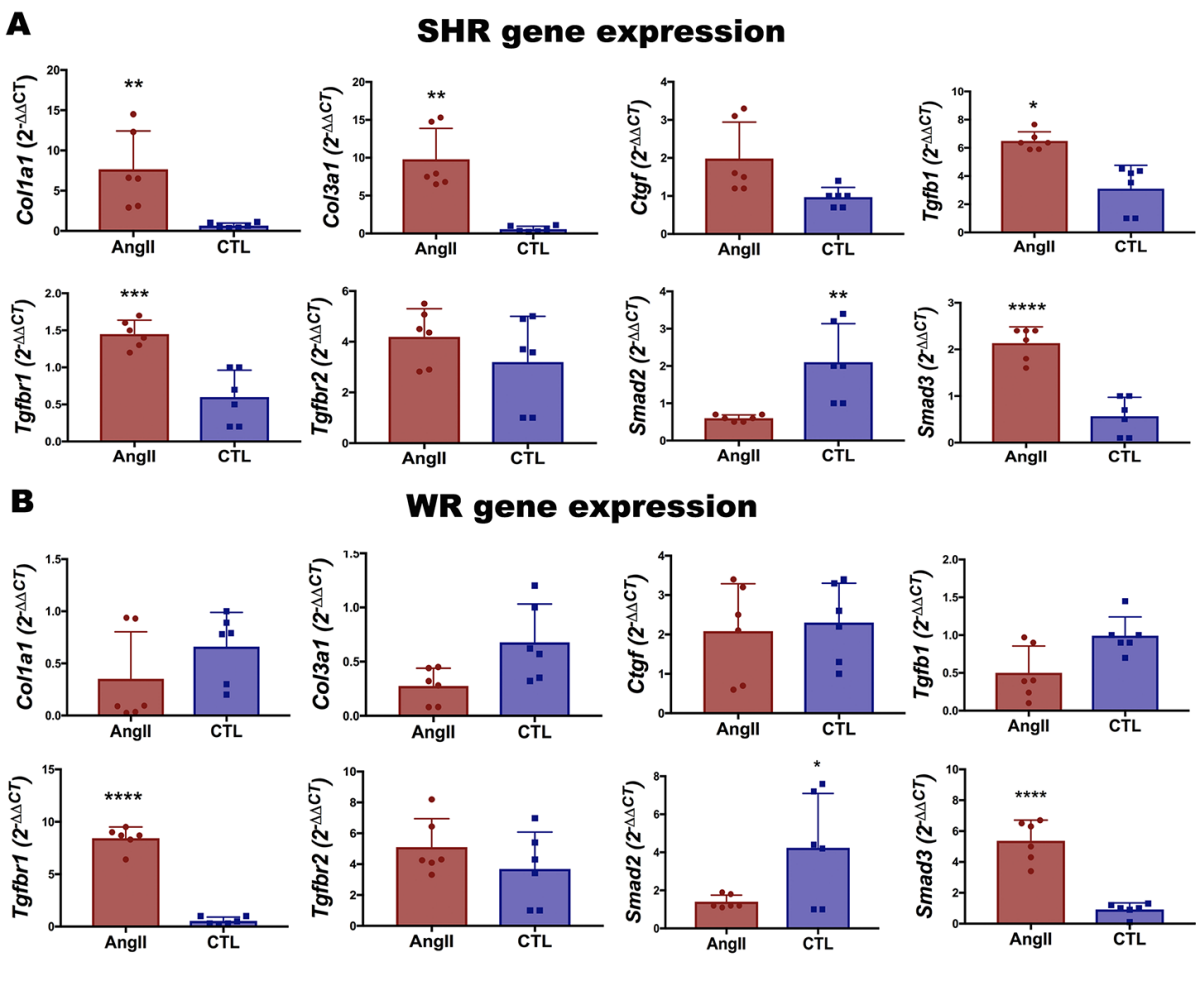
Fibrogenic gene expression profile in SHR and WR myoblasts after treatment with AngII. Expression of fibrogenic genes in primary culture of SHR (A) or WR (B) myoblasts (passage 4) by RT-qPCR, 24 h after treatment with AngII (100 nM). n = 6 per group. Bars represent mean and standard deviation. * p<0.05, **p<0.01, ***p<0.001, ****p<0.0001.

We investigated the fibrotic effect of AngII in SHR and WR myoblasts by RT-qPCR. AngII upregulated the fibrogenic genes *Col1a1*, *Col1a3*, *TGFbr1* and *Smad3* on SHR cells (Fig 3A), whereas only *Tgfbr1* and *Smad3* were upregulated on WR cells (Fig 3B). *Smad2* was downregulated on both cells in the presence of AngII (Fig 3A and 3B).

### Down-regulation of the AT1a receptor reduces fibrosis in the SHR skeletal muscle

**Fig 4:**
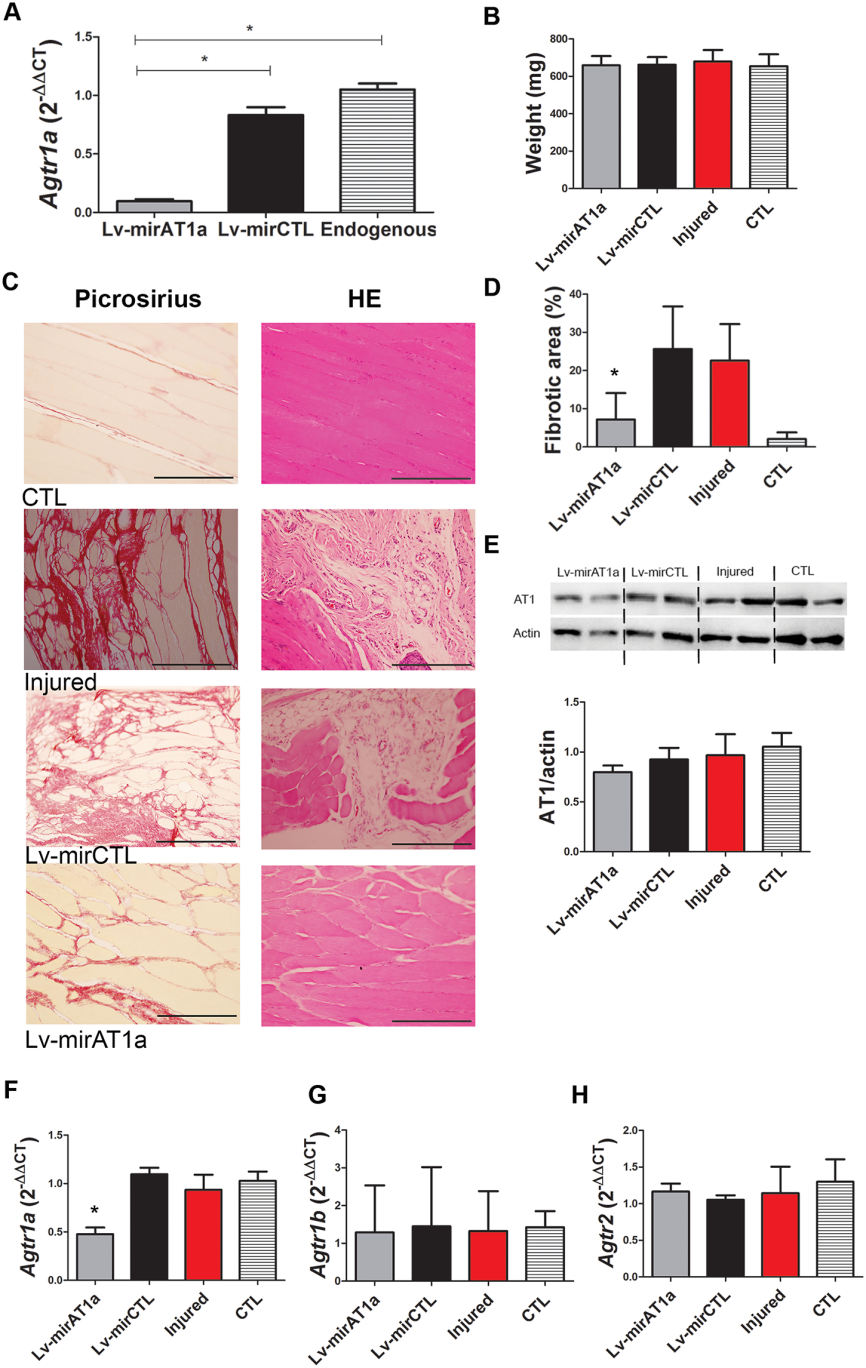
Effect of Lv-mirAT1a on laceration-injured skeletal muscle. (A) The L6 myogenic cell line was transduced with the Lv-mirAT1a or Lv-mirCTL lentivectors (MOI = 10), and the AT1a gene expression was evaluated 48 h later by RT-qPCR. (B) Lentivectors were injected into the TA muscle from SHR soon after LS lesion. Later (30 days), the animals were euthanized for TA muscle weight quantification, (C) histology (scale bar = 100 μm), (D) determination of the fibrotic area using data from Fig B, (E) expression of the AT1 protein gene by Western blot analysis, and (F) expression of the *Agtr1a*, (G) *Agtr1b* and (H) *Agtr2* genes by RT-qPCR analysis. n = 5 rats per group. Values represent mean and standard deviation. TA: *Tibialis anterior*; SHR: spontaneously hypertensive rat; CTL: SHR without injury; PR: Picrosirius Red; HE: hematoxylin-eosin stain; * p<0.05.

Initially, the efficiency of lentivectors to down-regulate the *Agtr1a* gene was assessed in rat myoblast L6 cells, which express the *Agtr1a* gene normally [47]. Lv-mirAT1a transduced L6 cells had a reduction in *Agtr1a* mRNA expression in comparison with non-transduced (91.0%) or transduced (88.5%) Lv-mirCTL (Fig 4A), showing high knockdown efficacy. No statistical difference between non-transduced and transduced with Lv-mirCTL groups was observed.

To assess the effect of Lv-mirAT1a in fibrosis, this lentivector was injected just after laceration and the TA muscles were analyzed four weeks after the treatment. No significant differences in muscle weight were observed among groups (Fig 4B). In histology, the injured rats or the treated with Lv-mirCTL showed an intense PR staining in a large area, whereas the group treated with Lv-mirAT1a showed a less stained area and a pattern similar to that of non-injured muscle (Fig 4C and 4D). Fibrosis was reduced after treatment with Lv-miAT1a, in comparison with the groups injured (16%) or treated (18%) with Lv-mirCTL (Fig 4D). In addition, muscle architecture of Lv-mirAT1a rats was more similar to that of non-injured rats, which was very different from the irregular muscle architecture as observed in rats injured or treated with Lv-mirCTL (Fig 4C).

No difference was observed among groups in AT1 receptor protein expression (Fig 4E). However, a significant reduction (53%) was observed in *Agtr1a* mRNA expression in the group treated with Lv-mirAT1a in comparison with that one of the non-injured group (Fig 4F). No difference was observed in the *Agtr1b* and *Agtr2* mRNA expressions between groups (Fig 4G and 4H).

### Gene expression analysis of myoblast primary culture after treatment with Lv-mirAT1a

**Fig 5:**
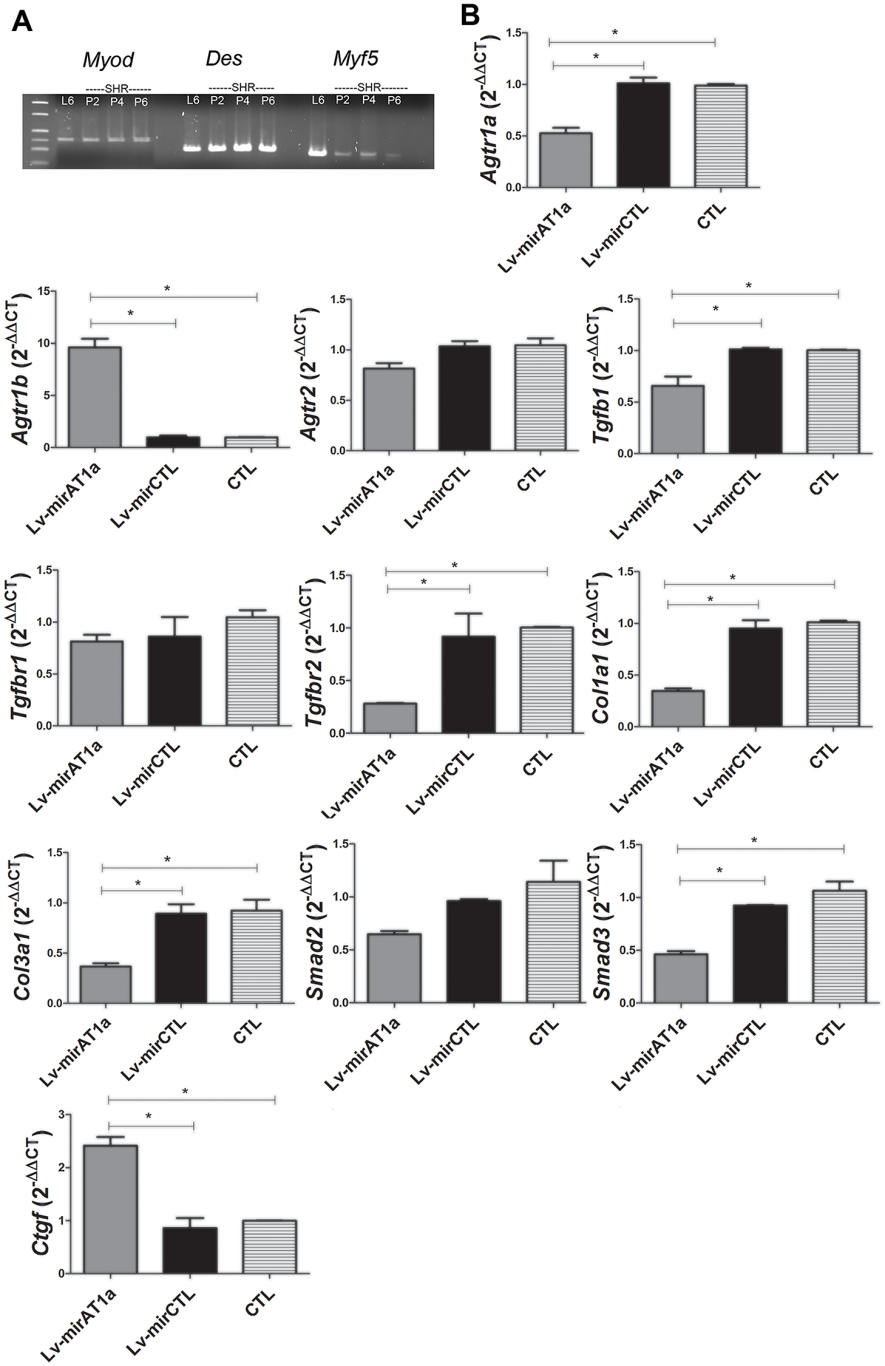
Gene expression analysis of myoblasts from SHR after treatment with Lv-mirAT1a. (A) Expression of myogenic genes in primary culture of SHR myoblasts from passages 2, 4, and 6. Cell line L6 was used as a control. (B) SHR myoblasts (passage 4) were transduced with the Lv-mirAT1a or Lv-mirCTL lentivectors, and gene expression was analyzed 48 h later by RT-qPCR. n = 3 rats per group. Bars represent mean and standard deviation. * p<0.05.

Finally, to investigate the genes regulated by the AT1a receptor, we established a primary culture of myoblasts from SHR neonates and the expression of myogenic markers (*Myod*, *Des*, and *Myf5*) was evaluated by RT-qPCR in different passages, using rat myoblast cell line L6 as a positive control. These cells were positive for the myogenic genes until passage 6 (Fig 5A).

These cells were then transduced with Lv-mirAT1a, and the gene expression profile was analyzed 48 h later (Fig 5B). The *Agtr1a* expression was reduced (50%) after transduction with Lv-mirAT1a as compared to the animals of the non-transduced group. In contrast, *Agtr1b* expression was increased after transduction with Lv-mirAT1a. Regarding *Agtr2*, no significant difference was observed between groups.

The *Tgfb1*, *Tgfbr2*, *Col1a1*, *Col3a1*, and *Smad3* genes related to fibrosis were down-regulated after transduction with Lv-mirAT1a (Fig 5B). The *Tgfbr1* and *Smad2* genes were also down-regulated, but the variations were not statistically significant. The *Ctgf* gene was the only one up-regulated after transduction with Lv-mirAT1a.

## Discussion

Angiotensin II plays a central role in blood pressure control via AT1, also binding to AT2 with similar affinity. However, several studies suggest that AT2 can antagonize AT1-mediated activities, in addition to having activities not related to AT1 signaling [48]. In rats, there are two AT1, AT1a, and AT1b subtypes, which have similar affinity for AngII. However, studies with AT1a and AT1b KO mice show that the block of AT1a gene affects much more blood pressure than of AT1b, showing the importance of AT1a in the blood control than AT1b [49,50]. These genes are expressed differently in tissues [51]. Since the ATR distribution in rat tissues is variable, we first investigated the expression pattern of angiotensin receptors in the skeletal muscle, using the heart as an experimental control due to the availability of data on angiotensin receptor gene expression in this tissue [52–54].

Our gene expression data show higher AT1 expression in the heart and the skeletal muscle of SHR as compared to WR (Fig 2) and absence of AT2 expression in the heart of both adult rats, which explains in parts the spontaneous hypertension in SHR and AT1 specificity in the heart. The AT2 receptor is highly expressed during embryogenesis, but such expression drops down after birth, and its expression is maintained after birth only in some tissues [55]. In addition, its expression can vary with age and pathophysiological conditions, thus leaving the literature data apparently inconsistent. For example: AT2 expression was seen in the heart of rat fetus by immunohistology, and its expression still could be detected 4 weeks after birth [56]; in addition, very low level of AT2 expression was detected by RT-PCR in the aorta of SHR, which increased 2-fold after long-term treatment with losartan [57]. Therefore, the absence of AT2 expression in the heart of 16-week old SHR and WR, as seen by two very sensitive methods (RT-qPCR and western blot), is not contradictory to other data but reinforce the idea that AT2 expression decreases with age in the heart of healthy rats.

Another significant difference between the heart and TA muscles of SHR is that AT1a is the unique form found in the heart, whereas presence of AT1b is about 3-fold higher than AT1a in the TA. As was commented above, AT1a is the main regulator of arterial pressure and AT1b contributes minimally [49,50], which is why the application of a miRNA specific to AT1a significantly decreased fibrosis, even though the expression of AT1b is much higher than AT1a in the TA muscle. Fibrosis reduction after AT1a knock-down is evident (Fig 4D and 4F), and the total amount of AT1 protein found 30 days after treatment with Lv-mirAT1a had the lowest level in comparison to the control groups (Fig 4E), although it was not significant statistically. As AT1 antibody does not distinguish between two AT1 subtypes, the AT1 band shown in the Fig 4E is the sum of AT1a and AT1b protein concentration. This fact should be the cause of a less impressive decrease of AT1 protein expression than its gene expression.

The larger extension of fibrosis as observed in SHR after laceration in comparison with WR (Fig 1D and 1E) and fibrosis reduction after treatment with Lv-mirAT1a (Fig 4D and 4F) were the first evidence of correlation between fibrosis and hypertension in the skeletal muscle, indicating that AT1-mediated hypertension should be the main cause of fibrosis in the skeletal muscle. Furthermore, the more expressive upregulation of fibrogenic genes in SHR myoblast than WR myoblast after treatment with AngII (Fig 3A and 3B) corroborates this assumption.

There are several evidences showing that interfering TGFβR with signaling by AT1 signaling is one of the mechanisms that organisms control fibrosis [12–14]. In order to verify the influence of AT1 on the expression of fibrotic genes, we used myoblasts isolated from SHR, once these cells are the main source to skeletal muscle regeneration after injury [42]. Our *in vitro* study show a clear influence of AT1 on the expression of *Tgfb1*, *Tgfbr2*, *and Smad3*, and fibrotic genes controlled by *Col1a1* and *Col3a1* genes (Fig 5), except for the profibrotic *Ctgf*, which was upregulated after mirAT1a treatment. CTGF is a pleiotropic factor that under normal circumstances is involved in angiogenesis and cellular differentiation, but in pathological conditions CTGF modulates, among others, wound healing and fibrosis [58–60]. Although CTGF regulates such large biological activities, its gene expression is modulated mainly by TGFß1 [61–63] but AngII can also induce TGFß1 expression independently [64]. Thus, although AT1 signaling and TGF-ß1 receptor signaling have cross-talk, the knockdown of AT1 with mirAT1a does not necessarily interrupt TGF-ß1 signaling and *Ctgf* expression. Therefore, it seems that skeletal muscle fibrosis in hypertensive organisms follows the same mechanism ones of the normotensive organisms, but more markedly.

Losartan, an angiotensin receptor antagonist, has been shown to be a good inhibitor of skeletal muscle fibrosis [14,65] and cardiac muscle fibrosis after injury [12,34]. Even losartan is a FDA-approved drug and is widely used to treat hypertension, its oral use to treat skeletal muscle fibrosis after injury can bring unnecessary undesired side effects, such as upper respiratory infections, dizziness, hyperkalemia, and other symptoms [36–38]. Knockdown of AT1 with Lv-miRNA or a similar vector system can avoid these side-effects since the vector is delivered locally. To maintain the injected vectors in the muscle, avoiding vector leaking and spreading, these lentivectors can be prepared with alginate hydrogel and injected directly into the muscle. Just one injection can last more than two months of transgene expression in the muscle [66], a sufficient time to heal the injured muscles, without targeting other tissues [39].

Collectively, our in vivo and in vitro studies indicate that hypertension promotes more skeletal muscle fibrosis, and AngII-AT1a signaling is the main pathway of fibrosis in SHR, and muscle fibrosis can be specifically treated with *in loco* injection of Lv-mirAT1a without affecting other organs. Our studies also indicate that the skeletal muscle injury in hypertensive people is prone to have more muscle fibrosis than in normotensive ones, thus contributing to the development of muscle specific gene therapy vectors to improve muscle healing after injury with less fibrosis.

## Supporting information

S1 Table List of all primers used in RT-qPCR experiments. (DOCX)

S1 File The ARRIVE guidelines checklist. (DOCX)
