# Supplementary material for: Reduction in skeletal muscle fibrosis of spontaneously hypertensive rats after laceration by microRNA targeting angiotensin II receptor
Source: PLoS One. 2017 Oct 23;12(10):e0186719. doi: 10.1371/journal.pone.0186719 (PMC5653346; doi:10.1371/journal.pone.0186719)
Supplement: S1 Table — (DOCX) [file pone.0186719.s001.docx]

**Table S1. List of all primers used in RT-qPCR experiments**

| **Genes** | **Forward primers (5’ to 3’)** | **Reverse primers (5’ to 3’)** |
| --- | --- | --- |
| *Tgfb1* | CCCCTGGAAAGGGCTCAACAC | TCCAACCCAGGTCCTTCCTAAAGTC |
| *Col1a1* | ATCAGCCCAAACCCCAAGGAGA | CGCAGGAAGGTCAGCTGGATAG |
| *Col3a1* | TGATGGGATCCAATGAGGGAGA | GAGTCTCATGGCCTTGCGTGTTT |
| *Ctgf* | CAGGCTGGAGAAGCAGAGTCGT | CTGGTGCAGCCAGAAAGCTCAA |
| *Smad2* | TGAGCTTGAGAAAGCCATCA | TGTGTCCCACTGATCTACCG |
| *Smad3* | CATTACCATCCCCAGGTCAC | CGTAACTCATGGTGGCTGTG |
| *Tgfbr1* | CTGCAATCAGGATCACTGCAA | GCAGACTGGACCAGCAATGAC |
| *Tgfbr2* | caagaacattactctggagac | acaggagcacatgaagaaggt |
| *Myod* | GCAAGCGCAAGACCACTAAC | GCCGCTGTAATCCATCATGC |
| *Myf5* | GAAGGTCAATCAAGCTTTCG | TGAAGCACATGCGTTTGATA |
| *Des* | GGAGGAAATCCAACTGAGAG | TCAAGTCTGAAACCTTGGAC |
| *Rn18S* | CATTCGAACGTCTGCCCTAT | GGCCTCACTAAACCATCCAA |
