# Supplementary material for: Reduction in skeletal muscle fibrosis of spontaneously hypertensive rats after laceration by microRNA targeting angiotensin II receptor
Source: PLoS One. 2017 Oct 23;12(10):e0186719. doi: 10.1371/journal.pone.0186719 (PMC5653346; doi:10.1371/journal.pone.0186719)

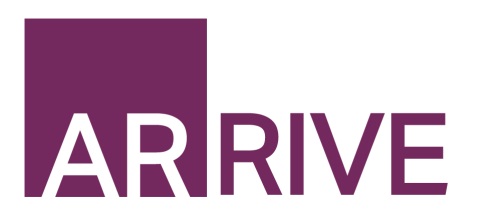


The ARRIVE Guidelines Checklist

Animal Research: Reporting In Vivo Experiments

Carol Kilkenny^1^, William J Browne^2^, Innes C Cuthill^3^, Michael Emerson^4^ and Douglas G Altman^5^

*^1^The National Centre for the Replacement, Refinement and Reduction of Animals in Research, London, UK, ^2^School of Veterinary Science, University of Bristol, Bristol, UK, ^3^School of Biological Sciences, University of Bristol, Bristol, UK, ^4^National Heart and Lung Institute, Imperial College London, UK, ^5^Centre for Statistics in Medicine, University of Oxford, Oxford, UK.*

|  | | ITEM | RECOMMENDATION | | Section/ Paragraph |
| --- | --- | --- | --- | --- | --- |
| 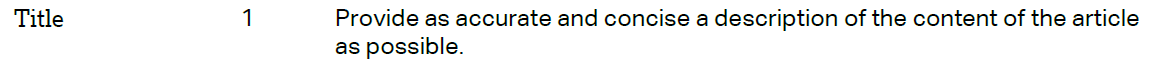 | | | | Page 1 |  |
| 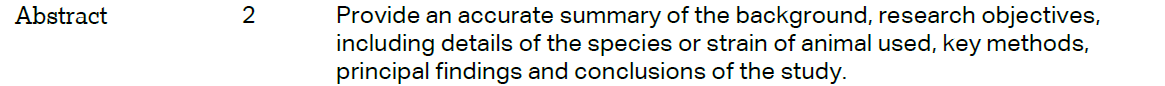 | | | | Page 2 |  |
| INTRODUCTION | | | |  |  |
| 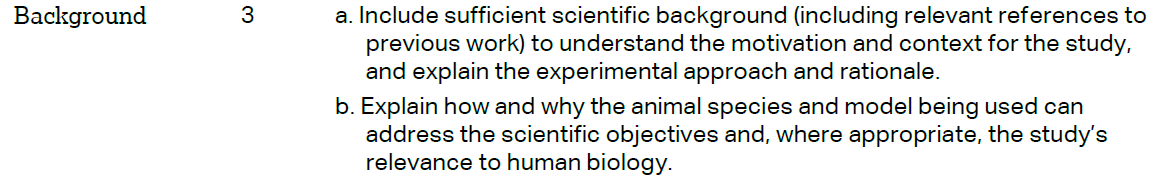 | | | | Page 4-5 |  |
| 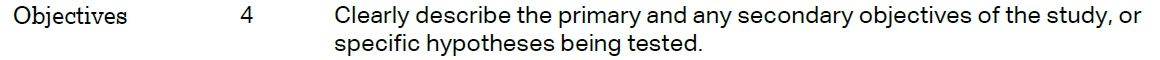 | | | | Page 5 1^st^ paragraph |  |
| METHODS | | | |  |  |
| 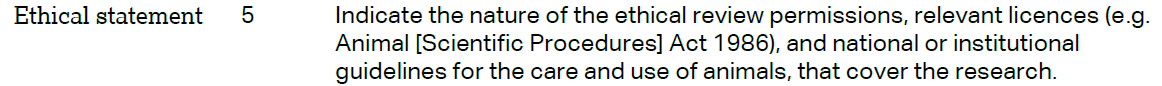 | | | | Page 5 2^nd^ paragraph |  |
| 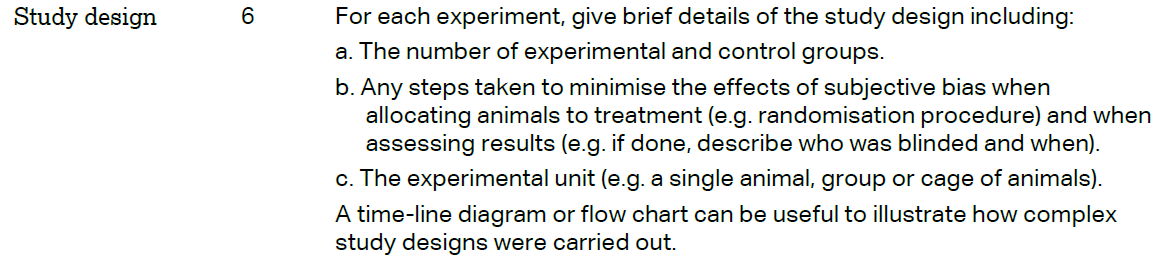 | | | | Page 5 3^rd^ paragraph |  |
| 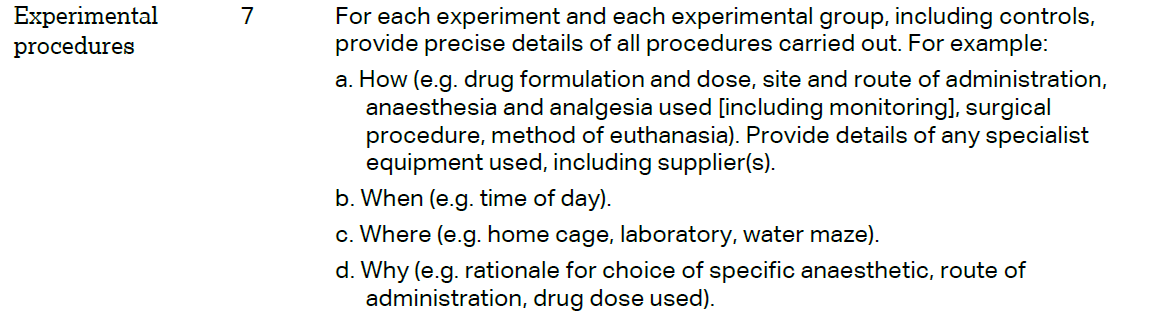 | | | Page 5-7 | |  |
| 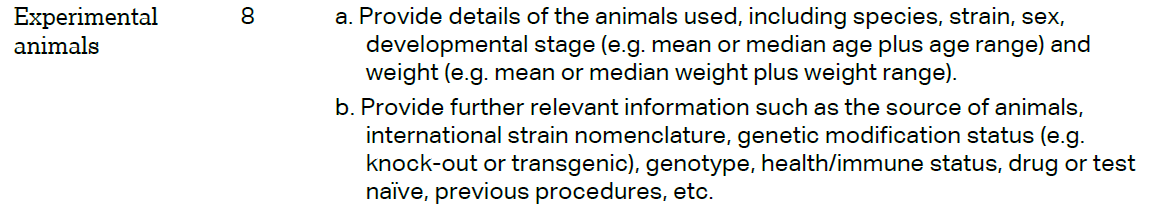 | | | | Page 5 and 6 |  |

The ARRIVE guidelines. Originally published in *PLoS Biology*, June 2010^1^

| 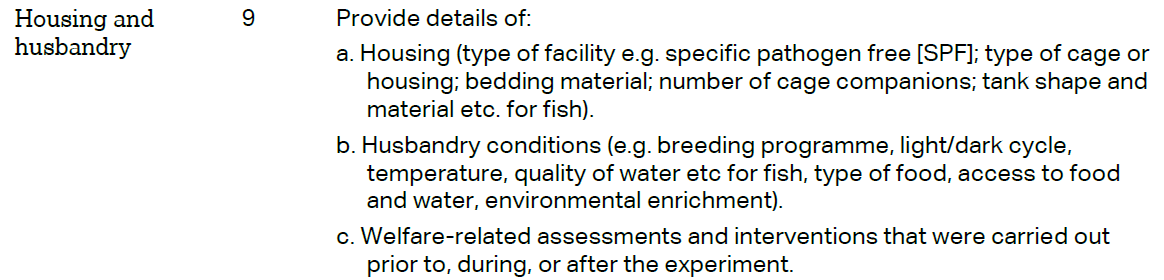 | Page 5 2^nd^ paragraph | |
| --- | --- | --- |
| 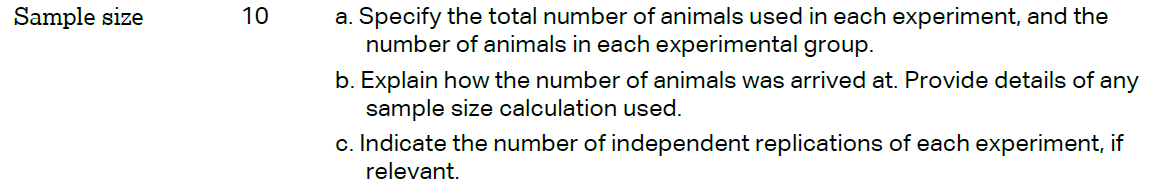 | Page 5 2^nd^ paragraph and Page 7 1^st^ paragraph  Figure 1 and 4 legend | |
| 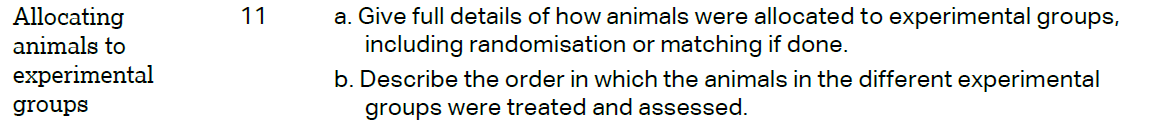 | Page 5-7 | |
| 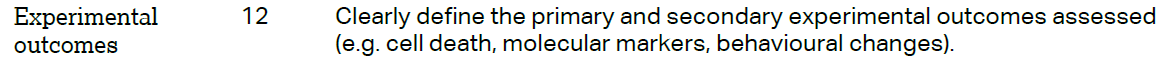 | Page 7-11 | |
| 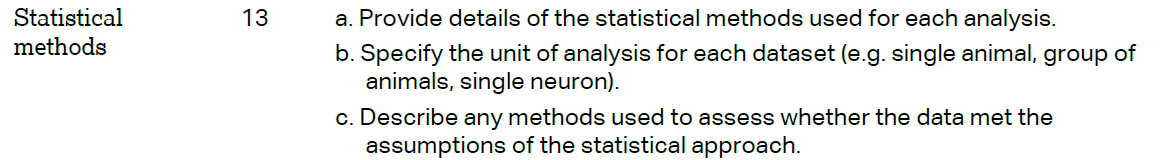 | Page 12 2^nd^ paragraph | |
| RESULTS |  | |
| 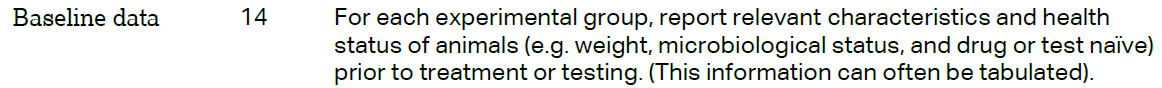 | Page 5 2^nd^ paragraph | |
| 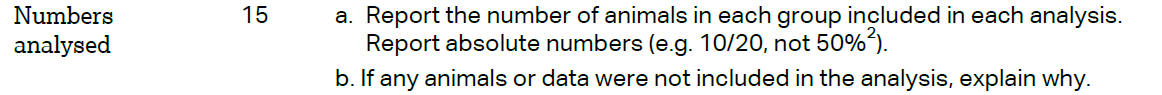 | Figure 1 and Figure 4 legend | |
| 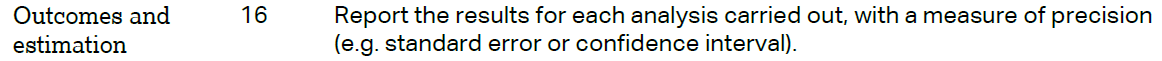 | Figure 1 and Figure 4 legend | |
| 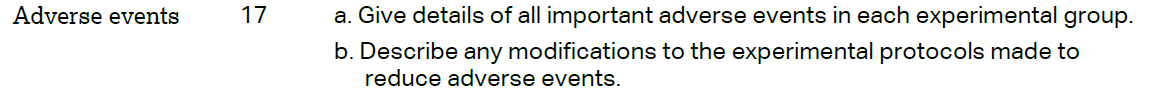 | N/A | |
| DISCUSSION |  | |
| 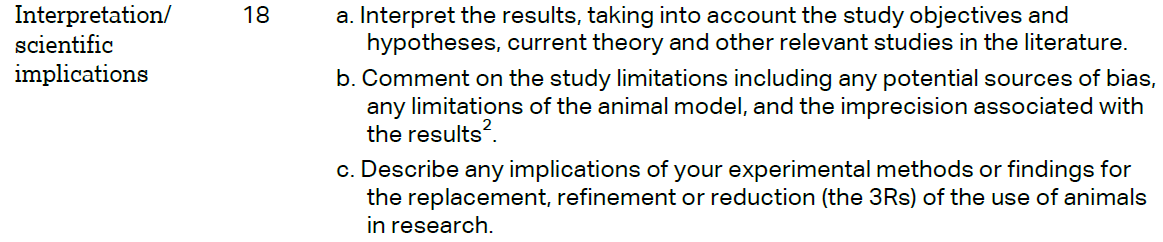 | Page 18 2^nd^ paragraph  Pages 19 and 20 | |
| 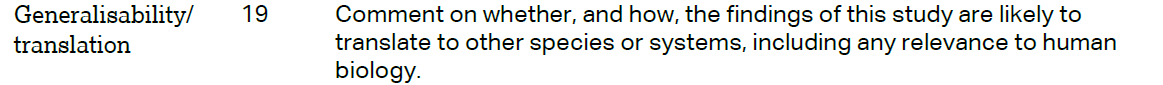 | Page 20 | |
| 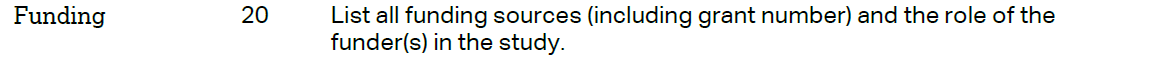 | | In the Financial Disclosure  Fundação de Amparo a Pesquisa do Estado de Sao Paulo (2015/20206-8); [www.fapesp.br](http://www.fapesp.br)  Conselho Nacional de Desenvolvimento Científico e Tecnológico (307044/2015-7); [www.cnpq.br](http://www.cnpq.br) |


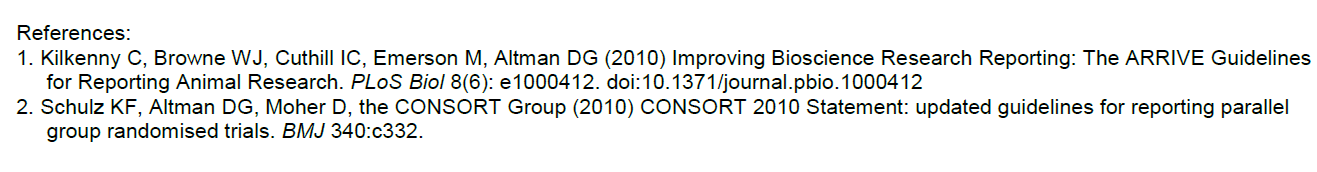

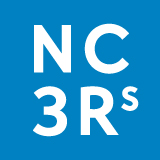

Supplement: S1 File — (DOCX) [file pone.0186719.s002.docx]
